# Air Pollution and COVID-19: The Role of Particulate Matter in the Spread and Increase of COVID-19’s Morbidity and Mortality

**DOI:** 10.3390/ijerph17124487

**Published:** 2020-06-22

**Authors:** Silvia Comunian, Dario Dongo, Chiara Milani, Paola Palestini

**Affiliations:** 1 Department of Biotechnology and Biosciences, University of Milano-Bicocca, 20126 Milan, Italy; s.comunian1@campus.unimib.it; 2 Égalité org, 00152 Rome, Italy; dario.dongo@icloud.com; 3 School of Medicine and Surgery, University of Milano-Bicocca, 20900 Monza, Italy; paola.palestini@unimib.it; 4 NeuroMi, Milan Centre for Neuroscience, University of Milano-Bicocca, 20900 Monza, Italy; 5 POLARIS Research Centre, University of Milano-Bicocca, 20900 Monza, Italy

**Keywords:** COVID-19, particulate matter, ACE2, inflammation, oxidative stress

## Abstract

Sars-Cov-2 virus (COVID-19) is a member of the coronavirus family and is responsible for the pandemic recently declared by the World Health Organization. A positive correlation has been observed between the spread of the virus and air pollution, one of the greatest challenges of our millennium. COVID-19 could have an air transmission and atmospheric particulate matter (PM) could create a suitable environment for transporting the virus at greater distances than those considered for close contact. Moreover, PM induces inflammation in lung cells and exposure to PM could increase the susceptibility and severity of the COVID-19 patient symptoms. The new coronavirus has been shown to trigger an inflammatory storm that would be sustained in the case of pre-exposure to polluting agents. In this review, we highlight the potential role of PM in the spread of COVID-19, focusing on Italian cities whose PM daily concentrations were found to be higher than the annual average allowed during the months preceding the epidemic. Furthermore, we analyze the positive correlation between the virus spread, PM, and angiotensin-converting enzyme 2 (ACE2), a receptor involved in the entry of the virus into pulmonary cells and inflammation.

## 1. Introduction

On 11 March 2020, the World Health Organization (WHO) declared the coronavirus pandemic: the Sars-Cov-2 virus (COVID-19) is a threat to the population’s health. Air pollution is also one of the greatest challenges of our millennium, and some early studies have highlighted a positive correlation between air pollution and the spread of the virus. Therefore, it is crucial to define which role the atmospheric particulate plays in the spread, morbidity, and mortality of the virus.

In this context, two hypotheses should be highlighted. First, COVID-19, similarly to other viruses, could also have an airborne transmission, and particulate matter (PM) could act as a carrier through the aerosol, conveying the virus and increasing its spread. Secondly, PM could instead have induced damage to the lung cells, increasing the inflammation state [1]. This rise of inflammation may increase the mortality rate and the severity of expression of the disease in the most polluted areas. The virus binds to the angiotensin-converting enzyme 2 (ACE2) receptor to enter the cell; ACE2 generates an anti-inflammatory peptide and is overexpressed to play its role in the case of inflammation from PM exposure, thus increasing the probability of COVID-19 entering the cells [2]. These hypotheses, which are not mutually exclusive, are an important starting point for future analyses aimed at explaining the positive correlation between the spread of COVID-19 and air pollution.

## 2. Airborne Transmission: Comparison between Sars-Cov-1 and Sars-Cov-2

Pathogens can reach the organism through various transmission mechanisms: ingestion (via the fecal-oral route), inhalation, inoculation, contact, iatrogenic transmission, and coupling. The most common route of transmission is the expulsion of pathogens through the respiratory system by infected subjects and the penetration into the receptive host by inhalation. The saliva droplets from the infected subject are usually large and, because of their weight, travel short distances before falling to the ground. In this case, the transmission is defined as transmission by close contact. This transmission is different from what occurs in the aerosol, which is a suspension of solid or liquid particles within a gas phase. The diameter of these particles is normally between 0.001 and 100 micrometers; thus, they are very small particles that sediment slowly and are easily conveyed by air currents (in this case, the transmission is called long distance transmission). As small viral particles are suspended in the aerosol, they can be transported by particles such as in the case of the avian flu virus that was found in large concentrations in the air after the dust storms that occurred in Asia even at a long distance from the outbreaks of origin [3].

The new coronavirus Sars-Cov-2 (COVID-19) emerged in late 2019 in the city of Wuhan in China and is now causing a global pandemic owing to its rapid spread. It is thus necessary to understand how the transmission takes place to limit its further diffusion. COVID-19, as the Sars-cov-1 virus, is mainly transmitted by inhalation of droplets, and with a smaller percentage by fecal–oral route, direct contact, and through pregnancy. Airborne transmission by aerosols in long distances has been proposed only as a possibility to be verified by Zhou Wang [4,5], assisted by a committee of experts who have addressed the epidemic of coronavirus pneumonia (COVID-19) in the city of Wuhan. 

COVID-19 belongs to the coronavirus (CoV) family, a large family of respiratory viruses that can cause mild to severe diseases, from the common cold to respiratory syndromes such as MERS (Middle East respiratory syndrome) and SARS (severe acute respiratory syndrome). The name coronavirus derives from the proteins that outline a crown shape and that are present on the surface of the virus. In 2003, SARS caused a minor, but equally significant epidemic, after which numerous studies were conducted about the route of transmission of the virus.

The spread of the virus was analyzed in closed environments such as in aircraft cabins. It was observed that SARS has three prevalent transmission routes: it spreads 21% by aerosol (long distance), 29% by close contact between individuals (droplets), and 50% by contact with surfaces (fomite route) [6].

A recent analysis proposed in The New England Journal of Medicine by van Doremalen and collaborators [7] investigates the stability of the new virus in aerosol and on surfaces by comparing it with Sars-Cov-1. The surfaces analyzed are plastic, stainless steel, copper, and cardboard. COVID-19 persists more on plastic and steel, while copper and cardboard are more inhospitable to the virus. From the results obtained, it is observed that COVID-19 remains in the aerosol for 3 h, slowly reducing the infectious capacity such as Sars-Cov-1. The half-life of Sars-Cov-2 and Sars-Cov-1 is similar in aerosol, with an average of about 1.1–1.2 h, indicating that the epidemiological differences between the two are probably owing to other factors. The virus persists on surfaces for days and in aerosol for hours [7].

Many studies highlight the association between airborne infections and ventilation systems in buildings, as in the case of other viruses such as measles, avian, and SARS. SARS spread in 17 major cities in 2003. In a city environment, people normally spend about 90% of their time indoors. A low ventilation rate, particularly in hospitals, increases the probability of virus contraction. In a building, the air circulates from one environment to another with turbulent flow that favors the establishment of microenvironments in which pathogens proliferate. Viruses are transported by the aerosol at a certain distance that depends on the design of the buildings in which they circulate. In this regard, a multidisciplinary study is needed to analyze this effect and determine the correct ventilation rate to be applied in closed environments to decrease the probability of spreading these viral pathogens [8]. This phenomenon has been analyzed in a Hong Kong hospital during the 2003 epidemic. In this analysis, it is observed that the droplets are moist, but begin to evaporate after release by decreasing their size and may become small enough to circulate in the air. In the Hong Kong hospital, the distribution of air was analyzed, and it was shown that an unbalanced air diffuser distribution led to the spread of the Sars-Cov-1 virus in the 8 A department, where a greater incidence of the number of infections occurred [9].

A similar study was conducted in Amoy Garderns, a group of residential buildings in Hong Kong that recorded an outbreak of SARS in 2003. The investigators analyzed the ventilation system and the air transmission of the virus, focusing on the residence buildings with the most elevated number of infections. This analysis further confirms the need for reviewing the internal air quality and ventilation designs in buildings, offices, homes, hotels, and hospitals [10].

The transmission modes of Sars-Cov-1 and COVID-19 are very similar, as COVID-19 also persists in the air, and thus it can be assumed that it is also transported at greater distances than those observed in close contact infection. Despite the similarities between the new and previous viruses, why has the new coronavirus displayed a higher spread rate in some areas?

## 3. Air Pollution is Present in the Aerosol

The air is composed of 78% nitrogen, 21% oxygen, and 1% other gases. Man is introducing into the air new components that can be harmful to the environment and human health. Air pollution is the result of the presence of gas and contaminating particles in the atmosphere. The gases include carbon monoxide (CO), nitrogen oxides (NOx), ozone (O_3_), sulfur dioxides (SO_2_), ammonia (NH_3_), and volatile organic compounds (VOCs), as well as some gaseous forms of metals. Particulate matter (PM) instead includes a mixture of compounds that can be grouped into five major categories: sulphates, nitrates, elemental carbon, organic carbon, and crustal materials (such as earth and ash).

PM, as defined by the Environmental Protection Agency (EPA), is a term that indicates the set of particles dispersed in the air for enough time to be diffused and transported. There are many sources of these particles. PM is classified as PM10 and PM2.5 based on a diameter of less than 10 micrometers or 2.5 micrometers, respectively [11].

PM are inhalable corpuscles that cause various damage to human health owing to their small size. Their toxicity is then increased as they can adsorb other substances such as polycyclic aromatic hydrocarbons (PAHs) and heavy metals. Hydrocarbons derive from oil and are higher in concentration during winter [12]. PAHs are hydrocarbons made up of two or more joined aromatic rings. Metals, on the other hand, are natural constituents of the earth’s crust. Metals that are toxic and tend to accumulate are mercury, chromium, cadmium, arsenic, lead, and uranium [13]. 

Air pollution is a global public health emergency that affects people of all ages in every part of the world. Nowadays, addressing ambient air pollution is the priority of the government, and the WHO issues air quality guidelines to defend the population in general, and the most vulnerable in particular [14]. 

Numerous epidemiological studies have shown the effects of air pollution on respiratory and cardiovascular systems. Short-term exposure to air pollution at higher levels reduces life expectancy by aggravating pre-existing respiratory and cardiovascular diseases [15]. Cardiovascular effects induced by PM are linked to particles’ deposition in the lungs, to their translocation through the air-blood barrier to extra-pulmonary sites, and to the resulting systemic inflammation [16,17]. Particles’ deposition rates are strictly linked to the particle size—smaller particles have the highest deposition efficiency. Numerous studies also highlight correlations between the effect of PM and male infertility [18] as well as neurodegenerative diseases [19,20,21].

In Lombardy (North of Italy), diesel combustion and solid biomass burning are responsible for 15% and 50% of the primary fine particles production, respectively [22]. Diesel exhaust is a complex mixture of solid, condensed (or liquid), and gaseous corpuscular fractions [23]. The solid fraction is represented by diesel exhaust particles (DEPs), with a bio-persistent core of about 10–30 nm in diameter [24]. These primary particles, composed of elemental carbon, can then agglomerate into larger soot aggregates with mean diameters of 60–100 nm [23]. The DEP surface can adsorb more than 300 chemical compounds, which include PAHs, aliphatic hydrocarbons, quinones, transition metals, and others [25]. Moreover, biomass burning (BB)-derived particles are obtained as a result of inefficient combustion that generates a multitude of partially oxidized organic chemicals, many of which have been associated with adverse health impacts [22]. However, DEP has been shown to be even more toxic than BB [26].

These particles are included in PM2.5 fraction, are less than 0.1 micrometers in size, and thus can be classified as ultrafine particles (UFPs). UFPs can show worse and different toxicity profiles in comparison with those of larger particles with the same composition, as their specific interaction with lung cells and their capability to translocate across the alveolar epithelial barrier [27]. Nonetheless, it cannot be excluded that systemic toxicity may be mediated also by PM or UFPs associated water-soluble components and/or biochemical mediators released in the lung and then translocated in blood circulation.

WHO has also indicated air pollution as responsible for a great risk for environment and health. Even more harmful to health is indoor air pollution in large urban areas. It is thus essential to have correct ventilation systems in closed environments as the particulate matter in the aerosol can also host pathogens such as viruses and bacteria, which can thus be easily transmitted.

## 4. PM Has a Role in Bacterial and Viral Transmission

Air is a vehicle through which microbial agents can move around the environment. Plants and cellular fragments, bacteria, fungi, viruses, parasites, and spores can be components of the bioaerosol [5]. Atmospheric PM would function as a carrier, or as a transport vector, for many viruses. Thus, PM may have increased the effectiveness of the virus spread in the aerosol as it creates a microenvironment suitable for its persistence [28]. PM10 and PM2.5 can be inhaled and, in addition to the polluting particles, the associated microorganisms are inhaled, too. Recent studies also indicate that microbial community composition and concentration are significantly affected by particle concentration and dimension [29]. The particles could also act as carriers, which have complex adsorption and toxicity effects on bacteria [30]. Certain particle components are also available as nutrition for bacteria and the toxic effect dominates in heavy pollution. The analysis of Franzetti et al. [31] of microbial community in PM10 and PM2.5 sampled in winter and summer in Milan (Italy) showed large seasonal variations in the microbial communities, with plant associated bacteria dominating in summer and spore forming bacteria in winter. The results obtained also indicate not only that the PM source can influence the presence of specific bacterial groups, but also that environmental factors and stresses can shape the bacterial community.

In addition, inhalation transports the particles deep into the lungs, especially those smaller than 2.5 microns (PM2.5 and UFPs), and this allows the virus to develop within the respiratory tract and to cause infections.

In 2007, Ciencewicki and Jaspers [32] reviewed different epidemiological and experimental studies, examining the association between and the effect of air pollutants and viral respiratory infections, though potential mechanisms mediating these effects are largely unexplored.

In Beijing, China, the composition of organisms in the air pollutants during a period with a high smog level was tested in a metagenomics analysis. Sequences of several pathogens including viral particles (0.1% in both PM10 and PM2.5) were identified. The quantity of these pathogens for the respiratory tract increases with the increase in the concentration of pollutants [33].

The avian influenza virus (H5N1) could be transported across long distances by fine dust during Asian storms [3], and the correlation between PM concentration and the virus spread has been observed in the case of the spread of measles in China. PM2.5 concentrations in 21 Chinese cities and the number of measles cases per day per city were studied. The analysis showed a positive correlation between those two factors. The 10 μg/m^3^ increase in PM2.5 per day is associated with a significant rise in the disease incidence [34]. A similar analysis of the children’s respiratory syncytial virus (RSV) spread in China in 2015 shows the same correlation. RSV is a virus that causes damage to the lungs and bronchitis. A positive correlation between the virus and PM concentration was observed. In fact, pollution increases the risk of RSV infection [35].

A 2018 analysis, carried out in the Po Valley, associates hospitalizations and the number of new RSV cases with PM10 concentration. The data for the analysis were collected by ARPA (Regional Environmental Protection Agency) in the region. The results of this analysis showed that, in the designated period, the highest number of hospitalizations occurred in Milan, the city that had reached the maximum concentration of PM10. This study also shows a correlation between short- and medium-term PM10 exposures (in particular, in the two weeks preceding hospital admission) and increased risk of hospitalization owing to RSV bronchiolitis among infants [36]. There are several mechanisms by which PM induces an increase in infected cases. A mechanism can be that the virus is bound to particles and transported, if favored by climatic conditions [34].

## 5. PM: Possible Role in COVID-19’s Contagion

Concerning the effect of PM pollution and the spread of viruses in the population, several recent studies have analyzed whether the different areas of the world with a high and rapid increase in COVID-19’s contagion were correlated to a greater level of air pollution. At present, there are three world areas where there has been a high number of people infected by COVID-19: China, where the pandemic started; Italy; and the USA, and the link between these countries is the very high level of air pollutants. This is the reason that recent studies have focused on these areas to find a possible correlation in air pollution and COVID-19’s contagion.

The investigation of this possible correlation should be analyzed at two levels: (a) the high level of air pollution over the last years, which has made the population more sensitive to COVID-19 (long-term exposition); or (b) the sensitivity to the virus, which is linked to the high level of air pollution in the period when the virus appeared (short-term exposition). It is known that chronic exposure to atmospheric PM contributes to increased hospitalizations and mortality, primarily affecting cardiovascular and respiratory systems, causing various diseases and pathologies including cancer [37]. Premature deaths due to acute respiratory diseases from such pollutants are estimated to be over two million per year worldwide [38,39].

To evaluate the long-term exposition hypothesis, Pansini and Fornacca [40] investigated the geographical expansion of the infection and correlated it with the annual indexes of air quality observed from the Sentinel-5 satellite orbiting around China, Italy, and the USA, using annual means, and analyzed different pollutant (PM10, PM2.5, sulfur dioxide, carbon monoxide, nitrogen dioxide, and ozone). The authors found positive significant correlations between COVID-19 infections and air quality variables in each country and concluded that higher mortality was also correlated with poor air quality, namely, with high PM2.5, carbon monoxide, and nitrogen dioxide values.

Regarding Italy, Fattorini and Regoli [41] and Conticini and collaborators [42] came to the same conclusions. It is known that the distribution of atmospheric pollutants (PM2.5, PM10, nitrogen dioxide, and ozone) in Italian regions during the last four years exceeds regulatory limits; in Northern Italy, particularly affected by COVID-19 contagions, the population has been constantly exposed to a chronic high level of air pollution. The conclusive data of these papers indicate that long-term air-quality significantly correlated with the cases of COVID-19 in up to 71 Italian provinces, providing further evidence that long-term exposure to air pollution may represent a favorable context for the spread of the virus.

Finally, Pansini and Fornacca [43], associating several annual satellite and ground indexes of air quality in China, Iran, Italy, Spain, France, Germany, United Kindom, and USA with the COVID-19 infection, found statistically significant positive correlations between the high level of air pollution and COVID-19 infections. In Italy, the correspondence between poor air quality and COVID-19 appearance and its induced mortality was the starkest yet.

Recently, Wu et al. [44], fitting zero-inflated negative binomial mixed models (corrected for confounders), estimated for the United States a relationship between long-term exposure to PM2.5 and mortality rates to COVID-19. They found statistically significant evidence. According to this analysis, a 1 ug/m^3^ increase in long-term exposure to PM2.5 is associated with a 15% increase in COVID-19 mortality rate. The results of this article suggest that long-term exposure to air pollution increases vulnerability to the occurrence of more severe COVID-19 results. These findings are in line with the known relationship between PM2.5 exposure and many of the cardiovascular and respiratory comorbidities that significantly increase the risk of death in COVID-19 patients [44]. Concerning the effect of the short-term PM exposition and the spread of viruses in the population, the position paper proposed by the Italian Society of Environmental Medicine (SIMA) considers PM as an important carrier that has contributed to the spread of COVID-19 [28]. The high, rapid spread of the COVID-19 virus in the Po Valley, which is also the foremost place of polluted air in Europe, could be related to the high concentrations of PM present in Lombardy, before the COVID-19 pandemic [45].

**Figure 1:**
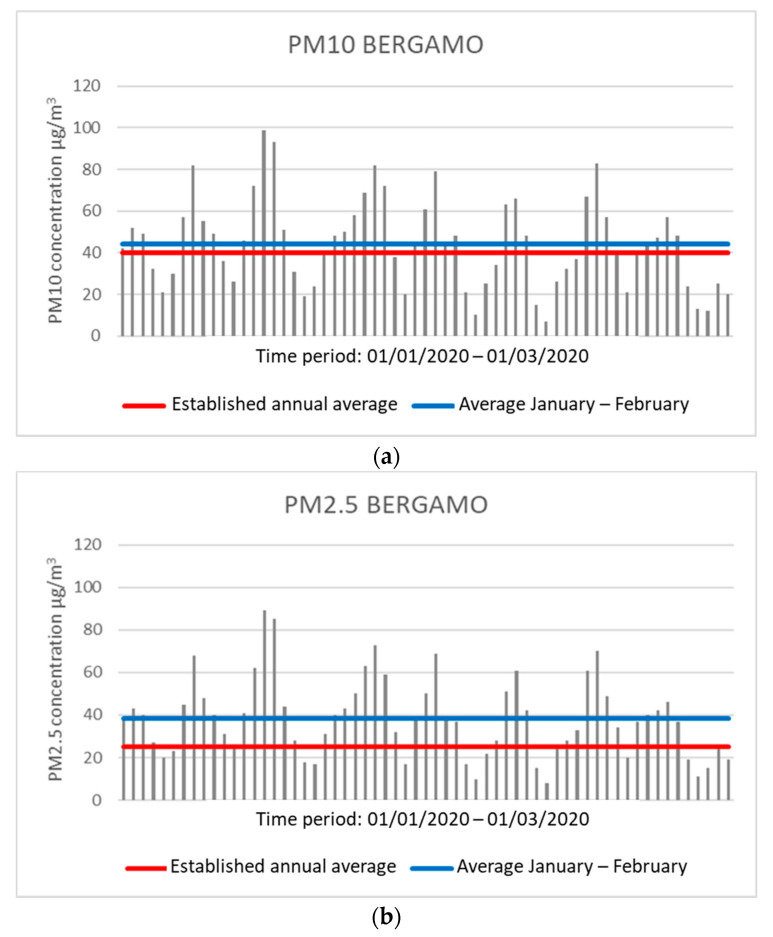
Graphs showing the concentration values of PM10 (**a**) and PM2.5 (**b**) in Bergamo. The red line indicates the expected regulatory limit, while the blue line indicates the average in the two months analyzed (analysis processed with data collected by ARPA (Regional Environmental Protection Agency)).

For example, Bergamo, one of the Italian cities with the highest number of infected, presents concentrations higher than the permitted annual average of PM10 and PM2.5 during the months of January and February 2020. The limits are 40 μg/m^3^ for PM10 and 25 μg/m^3^ for PM2.5. During these two months, a central unit of the city (via Meucci station) has detected an average daily concentration of 44.28 μg/m^3^ for PM10 and 38.31 μg/m^3^ for PM2.5, both values above the maximum limit set. The number of days in which daily concentration values beyond the allowed threshold was observed for PM10 and PM2.5 to be 33 and 44, respectively (Figure 1).

**Figure 2:**
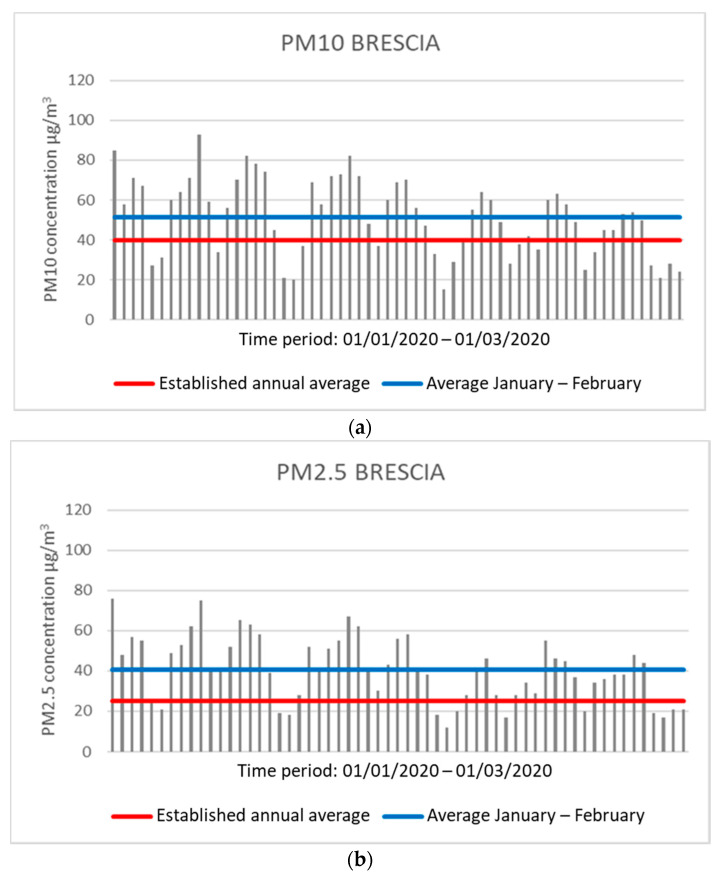
Graphs showing the concentration values of PM10 (**a**) and PM2.5 (**b**) in Brescia. The red line indicates the expected regulatory limit, while the blue line indicates the average in the two months analyzed (analysis processed with data collected by ARPA).

In Brescia, during the same period, an average of 51.49 μg/m^3^ for PM10 and 40.68 μg/m^3^ for PM2.5 was observed by the control unit located in Villaggio Sereno, all values above the threshold. There are 42 days for PM10 and 46 days for PM2.5 in which the daily concentration exceeds the average annual concentration allowed (Figure 2).

**Figure 3:**
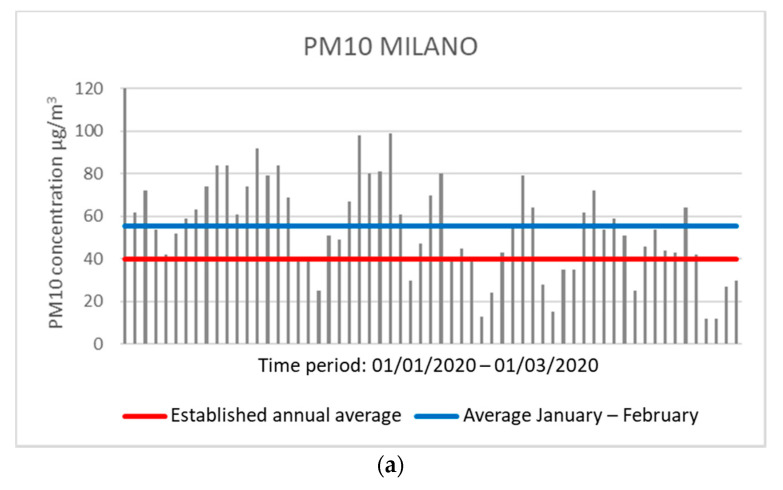

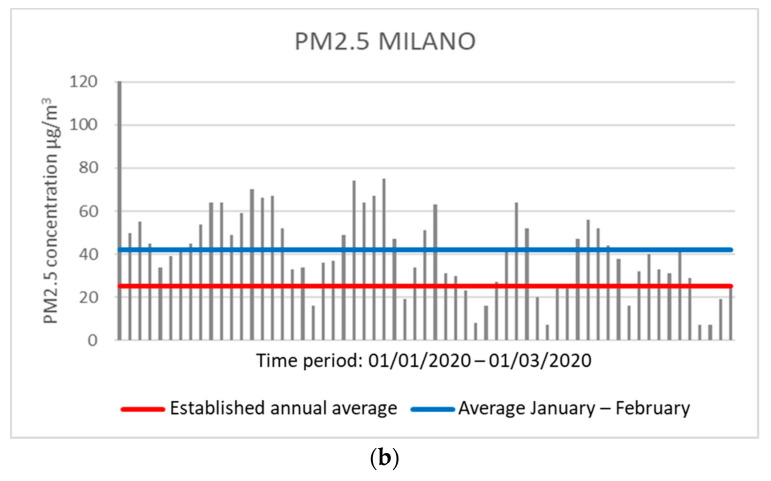
Graphs showing the concentration values of PM10 (**a**) and PM2.5 (**b**) in Milano. The red line indicates the expected regulatory limit, while the blue line indicates the average in the two months analyzed (analysis processed by data collected by ARPA).

The same observation can occur in Milan with the control unit in the Città Studi area, which showed an average concentration of 55.59 μg/m^3^ for PM10 and 42.18 μg/m^3^ for PM2.5 over the two months. Moreover, in Milan, 45 and 49 days were observed (for PM10 and PM2.5, respectively) with daily concentration values above the annual average allowed (Figure 3).

**Figure 4:**
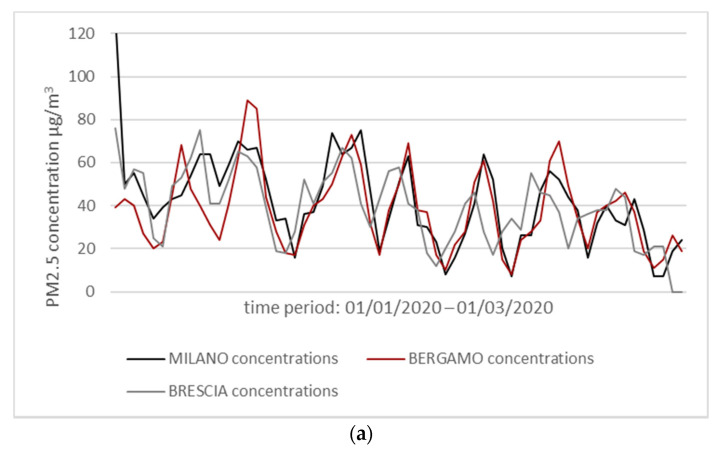

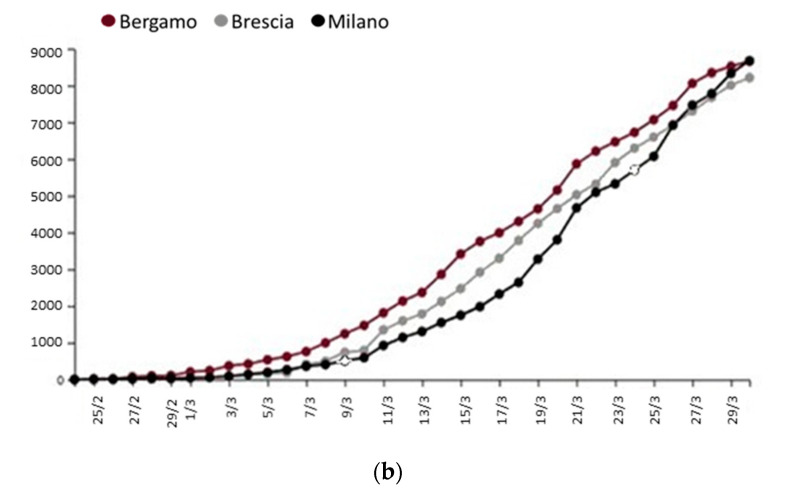
(**a**) Graph showing a comparison between PM2.5 concentrations in Bergamo, Brescia, and Milan during January and February 2020. (**b**) COVID-19 positive’s growth in Bergamo, Brescia, and Milan from late February to late March 2020 (generated and modified from [46]).

From the analysis of the data collected by ARPA (www.arpalombardia.it/Pages/Aria/qualita-aria.aspx) in the Italian region during the months of January and February, concentrations of PM10 and PM2.5 were measured above the limits established by the country’s regulatory parameters. In these same provinces, there is a high and rapid increase in COVID-19’s contagion (Figure 4).

This evidence was confirmed by different studies. Frontera et al. [47], analyzing air quality in Italy and China, in the period of maximum COVID-19 virulence, found that PM2.5 and nitrogen dioxide levels were particularly high, and Marteletti and Marteletti [48] arrived at the same conclusions. According to recent SIMA analysis, these authors speculate that the atmosphere, rich of air pollutants, together with certain climatic conditions, may promote a longer permanence of the viral particles in the air, as a carrier, thus favoring an “indirect” diffusion.

Setti et al. [49] showed that a high frequency of PM10 concentration peaks (exceeding 50 μg/m^3^) results in a spread acceleration of COVID-19, suggesting a “boost effect” for the viral infectivity. They also found significance differences both in PM10 exceedances and COVID-19 spreading between Northern and Southern Italian regions and placed a focus on Milan and Rome.

Zhu et al. [50] analyzed the relationship between concentrations of six daily measured air pollutants (PM10, PM2.5, sulfur dioxide, carbon monoxide, nitrogen dioxide, and ozone) and confirmed COVID-19 cases in 120 cities in China between 23 January 2020 and 9 February 2020 (short-term exposition). The authors examined the effects of the six air pollutants using six separate models to reduce collinearity, as some of these pollutants were highly correlated. They found significant positive associations of PM2.5, PM10, carbon monoxide, nitrogen dioxide, and ozone with COVID-19 confirmed cases, while sulfur dioxide was negatively associated. The authors conclude that there is a statistically significant relationship between short-term exposure to higher air pollution and an increased risk of COVID-19 infection.

The hypothesis that the atmosphere, rich of air pollutants, together with certain climatic conditions may promote a longer permanence of the of viral particles in the air, thus favoring an “indirect” diffusion in addition to the direct one (individual to individual), has been postulated in a recent letter to the Editor of Journal of Infection [47]. Considering a period of latency owing to the incubation of about 14 days, a positive correlation can be observed between the PM concentration data and the number of infections (Figure 4). The high concentrations of PM during January and February could have increased the virus spread rate and speed in late February/early March in Lombardy more than in other Italian regions.

Lastly, another point to consider is the climatic conditions that could also have played an important role in increasing the contagion. In fact, the main atmospheric circulation pattern during February 2020 was characterized by an anomalous anticyclonic system over the western Mediterranean basin, centered between Spain and Italy, and lower pressures over Northern Europe. This climatic condition, which produces dry conditions over southwestern Europe, may have provided optimal meteorological conditions for the virus propagation, both indoors and outdoors, in addition to the direct and indirect contact and short-range droplets [51]. Thus, PM may have played a carrier role for COVID-19. However, this remains one of the hypotheses, as the positive correlation between pollution in Lombardy and in other countries and the outbreak of the epidemic could also be the result of other reasons. In fact, it is known from both in vitro and in vivo data and from epidemiological studies that lasting exposure to airborne pollutants induces a state of chronic lung inflammation [12,32,52,53,54,55,56].

## 6. PM Damages Pulmonary Cells Inducing Inflammation and Oxidative Stress

Numerous epidemiological studies have noted an association between pollution levels and hospitalizations for many reasons including respiratory diseases. An increase in the mortality rate of several viral diseases has also been observed. So, in addition to analyzing the PM role in transmission, it is important to understand how exposure to pollutants can increase the susceptibility and severity of these diseases. As already discussed, PM has small dimensions and, for this reason, it can be inhaled. The repeated inhalation of these particles causes damage to lungs’ health. Long-term exposure to particulate matter can induce systemic damage [32]. Exposure to pollution has also been linked to the high mortality of the Sars-Cov-1 virus. Cui et al. [57], using the air pollution index (API), showed that, in the regions where this index was high, there was double the chance of death than the areas with a low API index. Similarly, in areas with a moderate API index, there was an 84% probability of increased risk of death from SARS. The authors conclude that lasting exposure to PM would increase the virus mortality rate.

After having demonstrated the positive correlation between viral infections in the respiratory tract and exposure to PM, it is necessary to analyze the mechanism by which exposure to these agents can impact the subject’s susceptibility and immune response to infection. As respiratory tract cells are the first target of PM, as well as the first target of respiratory viruses, pathogens will invade already compromised cells if subjects are exposed to PM for a long time. Two mechanisms have been demonstrated to be induced in lungs after PM exposure, both in humans and experimental models [58]:

1. Oxidative stress; exposure to these pollutants induces the production of free radicals that induce damage the cells.
2. Inflammation; PM induces the activation of the immune response and thus the cell enters in an inflammatory state.

**Figure 5:**
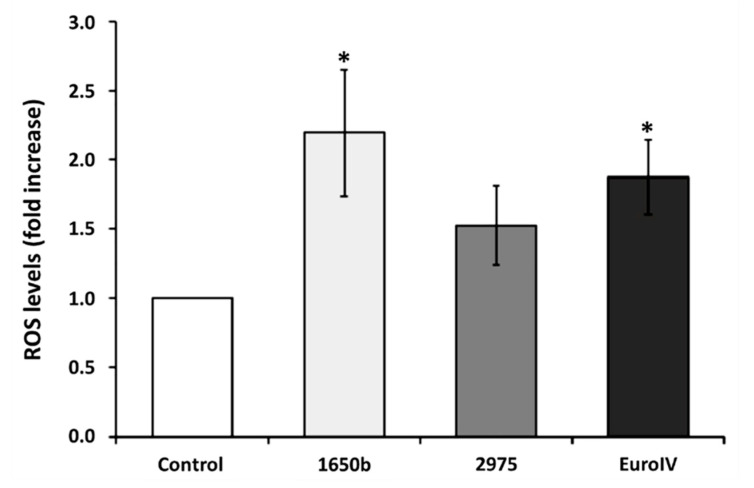
Image showing the increase in the level of reactive oxygen species (ROS) owing to 90 min exposure to 5 μg/cm^2^ of diesel pollution, detected by a fluorescent probe. * Statistically significant respect to control according to unpaired *t*-test, *p* < 0.05. Reproduced with the permission from [53].

Begalli et al. [53] analyzed the effect of different DEPs on lung and vascular cells in vitro. The analysis highlights the increase in ROS (reactive oxygen species) a marker of oxidative stress (Figure 5).

**Figure 6:**
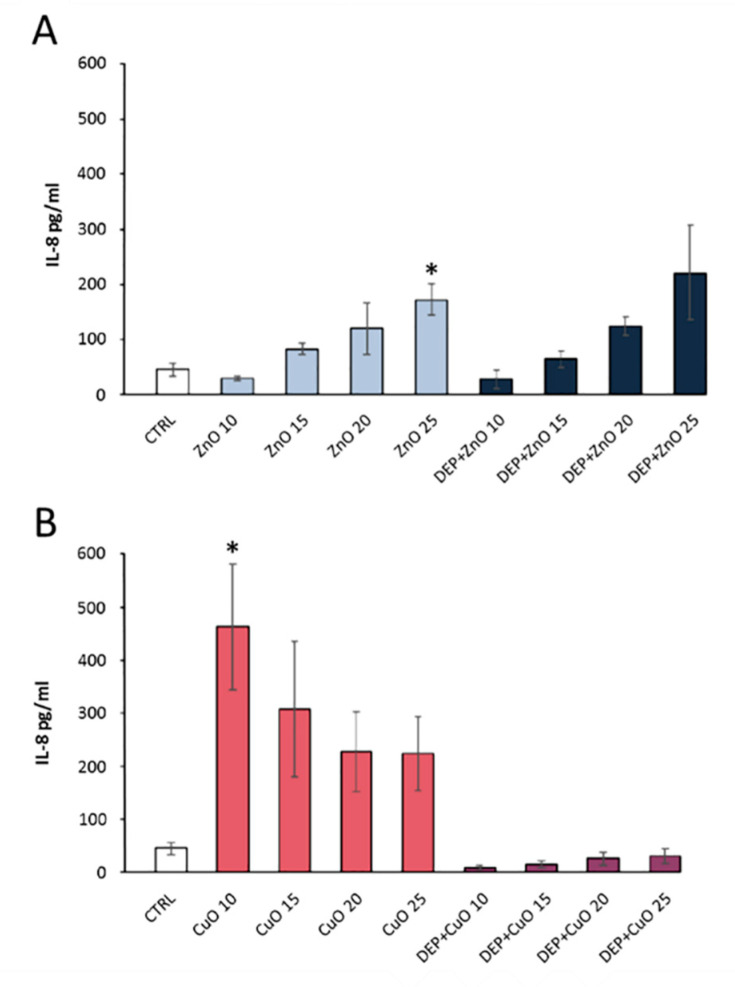
Analysis of the pro-inflammatory interleukin (IL)8 cytokine release in A549 cells after exposure to ZnO (**A**) and CuO (**B**) nanoparticles alone or in combination with diesel exhaust particles. Statistically significant according to the unpaired t test; *p* < 0.05. * Reproduced with the permission from [52].

DEP is rich in many metal oxides toxic to humans. An analysis on lung cells A549 has shown a significant increase in IL8, an interleukin used as an indicator of the pro-inflammatory response following exposure to pollutants (Figure 6) [52]. 

**Figure 7:**
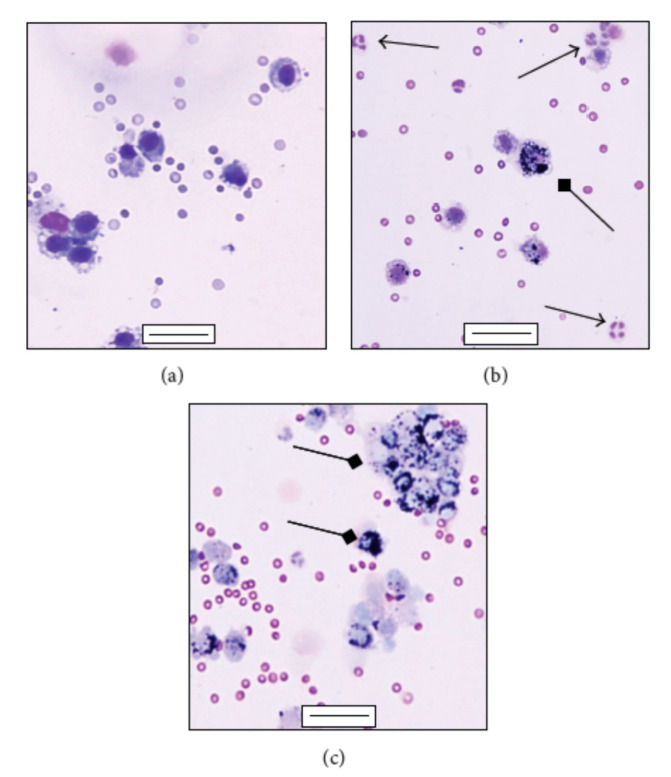
Differential staining of mouse bronchoalveolar lavage fluid (BALf) cells after PM1 intratracheal instillation. (**a**) Alveolar macrophages in the BALf collected 24 h post instillation from sham; (**b**) and (**c**) alveolar macrophages engulfing particles (square arrows) and infiltration of polymorphonuclear leukocytes (PMNs) (arrows) in the BALf collected 24 h after the last intratracheal instillation from PM1-treated mice. (**a**–**c**) bars = 50 µm. Reproduced with the permission from [56].

In fact, it has been observed that there are numerous mechanisms acting at the level of inflammation. For example, another mechanism to consider is the ability of pollutants to have effects on immunity by modulating the antiviral response of exposed subjects. A fundamental role of the inflammatory response is given by macrophages, cells that can incorporate foreign particles, including microorganisms, into their cytoplasm and destroy them (Figure 7) [56]. However, some studies have shown that exposure to pollution can reduce the phagocytosis ability of macrophages, which thus will not be able to properly inactivate the viruses [54].

Summing up, the exposure to polluting agents alters the immune response of the lung cells and induces an increase in oxidative and inflammatory stress. This cellular condition facilitates the attack of viruses and increases the severity of viral infections in exposed subjects. For example, pneumonia, often of viral origin, increases as a result of episodes of high PM10 pollution. A 1999 study already investigated how PM10 alters respiratory tract inflammatory responses to the syncytial virus (RSV), a frequent cause of viral pneumonia in infants and the elderly. It has been observed that, at high levels of PM10, the response to the virus is reduced because the immune response to both the virus and PM10, simultaneously detected, is less effective than the single immune response focused on defense against the RSV [59].

The Po Valley in northern Italy, where Milan is located, represents one of the most polluted areas of the country for both the high number of industries present and for its particular geographical conformation as it is enclosed between the Alps and the Apennines. During 2010, in Milan Torre Sarca, PM10 and PM2.5 samples were collected, and administered to mice for the purpose of analyzing the damage due to exposure to these particulates in the lung. The microbiological analysis revealed the presence of pathogens adsorbed to the particles [31], and in alveolar cells and in the lungs, PM activated strong inflammatory responses. In fact, high levels of pro-inflammatory cytokines such as tumor necrosis factor (TNF)-alpha and IL6 have been found [12]. It should be noted that IL6 is responsible for the inflammatory storm that occurs in the most serious cases of patients with COVID-19.

PM10 performs a toxic action on the lung parenchyma; the analysis of the content of BALf, a fluid resulting from mouse bronchoalveolar lavage, has shown an increase in cytotoxicity markers. PM also contains metals with a very high cytotoxic effect on cells. Prolonged or persistent exposure to PM is supposed to exert heavy adverse effects on cell homeostasis mediated by a direct particles cell interaction within the district that PM reaches (lung, myocardial, or even neuronal tissues), or by the induction of a chronic inflammation, resulting in a general systemic inflammation and sustained oxidative stress status [59].

A systemic spreading of lung inflammation in PM10sum-treated mice has been related to the increased blood total cell count and neutrophils percentage, as well as to increased blood myeloperoxidase (MPO). The blood–endothelium interface activation has been confirmed by significant increases of plasma endothelin 1 (ET-1) and sP-selectin. Furthermore, PM10sum induced heart endothelial activation and PAH metabolism, proved by increased ET-1 and cytochrome P450 1B1 (Cyp1B1) levels. Moreover, PM10sum causes an increase in brain heme oxigenase 1 (HO-1) and ET-1. These results state the translocation of inflammation mediators, ultrafine particles, lipopolysaccharide (LPS), and metals associated to PM10sum, from lungs to bloodstream, thus triggering a systemic reaction, mainly involving the heart and brain. These results provided additional insight into the toxicity of PM10sum and could facilitate shedding light on mechanisms underlying the development of urban air pollution related diseases [55,56].

Another analysis showed that exposure to PM2.5 also determines a variation in the expression of some genes; the effect of PM acts systemically on the human body. Sancini et al. [60] found that ET-1, heat shock protein 70 (Hsp70), Cyp1A1, Cyp1B1 and Hsp70, HO-1, and MPO each increased within lung and heart of PM2.5win-treated mice. The PM2.5win exposure had a strong impact on global gene expression of heart tissue (181 upregulated and 178 down-regulated genes), but a lesser impact on lung tissue (14 up-regulated genes and 43 downregulated genes). Therefore, prolonged exposure to high quantities of PM is a significant threat to people, especially the elderly, who may be apparently healthy, but with compromises in the lung and cardiovascular tissue [60].

Recently, Farina et al. [26] demonstrated in vivo the activation of inflammatory response (COX-2 and MPO) in both respiratory and cardiovascular systems, after exposure to UFPs (DEP or BB, Biomass). Exposure to DEP also results in increases in pro- and anti-oxidant (HO-1, iNOS, Cyp1b1, Hsp70) protein levels, although stress persists only in cardiac tissue under repeated instillations. Statistical correlations suggest that stress marker variation was probably the result of soluble components and/or mediators’ translocation from the first deposition site. This mechanism appears more important after repeated instillations, as inflammation and oxidative stress endure only in the heart. In summary, the chemical composition of UFPs influenced the activation of different responses mediated by their components or pro-inflammatory and pro-oxidative molecules, indicating DEP as the most damaging pollutant in the comparison [26]. Studies have shown that patients with systemic diseases have an increased risk of developing severe forms of COVID-19 infection [61]. Therefore, it cannot be excluded that the virus would be facilitated in the action on cells already in deficit. This cell’s deficit would lead to more complex prognoses when there are damages to the lung and heart also owing to exposure to polluting agents.

## 7. COVID-19 and Inflammation

Coronaviruses can generate mild or very serious pathological responses. When these viruses attack the lower part of the respiratory tract, they cause serious respiratory damage, which can be fatal. Inflammation is an indispensable component of the response of our immune system. However, already for Sars-Cov-1, it was observed that, in patients with a worse course of the disease, there is a “cytokine storm”, an excessive and dysregulated response [62].

The new COVID-19 virus can also lead to several pathological effects, from milder ones, like colds and fever, to more severe ones, like pneumonia, that can get worse in acute respiratory distress syndrome (ARDS), a type of respiratory failure responsible for the accumulation of fluid in the lungs and excessive reduction of oxygen in the blood. Ten percent of COVID-19 cases can come to this pathological condition in which mechanical ventilation is involved and, in some cases, lead to death. The course of the disease is more complex for the elderly and for patients with other concurrent pathologies [63].

In the blood of the patients with severe symptoms of COVID-19, very large quantities of IL6 are detected, a marker of the inflammatory state. An inflammatory storm is observed; the immune response of the infected cells is to release cytokines that signal inflammation, but an excess of these pro-inflammatory signals can be harmful to the cells of the pulmonary epithelium [64]. 

The Italian Medicines Agency (AIFA) has authorized the first clinical trials to use drugs already available for treatment in COVID-19. One approach is to lower the inflammation generated by a too strong immune response. In fact, among these trials, many concern anti-inflammatory drugs aimed at quenching the inflammatory storm. For example, Tocilizumab is a monoclonal antibody that binds to the IL6 receptor by deactivating it. Therefore, blocking the IL6 transduction pathway, the inflammatory state could be decreased [65]. There are some predispositions to the inflammatory storm owing to the presence of other pathologies. Patients with a particular pathogenesis start from higher inflammatory cytokine levels and the advanced age unbalances the body towards a pro-inflammatory state. Another option is that may there are some genetic predispositions. 

To these hypotheses, we could add a predisposition due to PM exposure. We observed that one of the PM mechanisms of action on cells is to trigger an inflammatory state, with the production of interleukins such as IL8 or IL6. We could thus assume a predisposition by the subjects most exposed to PM over time to a manifestation of this storm of cytokines following the COVID-19 infection, and thus a more complicated course of the pathology. This hypothesis could explain the positive correlation between COVID-19, the PM concentration, and the high mortality rate in some polluted areas, such as in Lombardy.

The analysis of Conticini et. al. [42] of March 2020 proposes the correlation between air pollution and COVID-19 lethality, which varies in the different regions in Italy; in Lombardy and Emilia Romagna on 21 March 2020, it was 12%, while in the rest of Italy, it was 4.5%. Exposure to polluting agents makes the inhabitants of these areas more prone to developing severe respiratory conditions and it would be a predisposition to add as a co-factor [42].

## 8. Relationship between COVID-19, PM, and ACE2

COVID-19 is characterized by the presence of spike proteins on its crown, which allow it to bind to the angiotensin-converting enzyme 2 (ACE2) receptor present on the cells. The virus enters the cells by endocytosis, infecting them. ACE2 is a membrane enzyme found in the lungs, arteries, heart, kidney, and intestines’ cells, and it regulates blood pressure by catalyzing the cut of the vasoconstrictor peptide, angiotensin 2, into angiotensin 1–7, which is instead a vasodilator and anti-inflammatory molecule [66]. It is hypothesized that, because increasing age leads to an increase in blood pressure, as a compensatory response, our body expresses a greater number of ACE2 on cell membranes to respond to this effect. However, this increase in expression also heightens the targets that allow access to the COVID-19 virus. Some hypertensive drugs increase the expression of ACE2, so this correlation should be verified.

ACE2 also has another task, as the angiotensin 1–7 peptide also has an anti-inflammatory function. It has been observed that the activation of ACE2 can reduce the severity of lung damage from hyperoxia by inhibiting the inflammatory response and oxidative stress. ACE2 can in fact inhibit the intracellular signal of nuclear factor kappa-light-chain-enhancer of activated B cells (NFKB) (pathway that activates the inflammatory response) and activate that of nuclear factor erythroid 2-related factor 2 (NRF2) (pathway that activates the anti-inflammatory response), as a protective mechanism against ROS [67]. Thus, an increase in ACE2 increases the probability of attack by COVID-19, but on the other hand, the virus, by binding to ACE2, blocks its activity, which instead seems to be very important for the immune defense and protection against inflammation that we have seen to be the major cause of deaths from COVID-19. A lasting exposure to PM2.5 triggers oxidative stress and inflammation, and the two pathways activated are those mentioned above: Nrf2 to respond to oxidative stress and NFKB, which instead stimulates the inflammatory state [68].

In 2018, Lin and coauthors [2] saw that exposure to PM2.5 induces acute lung injury (ALI) in mice, leading to an increase in inflammation with an increase in cytokine levels. The effects of PM2.5 were observed in both wild type (WT) and in ACE2 (knockout) mice. It was observed that, while in WT mice, there was an improvement in lung damage after a few days, in mice lacking ACE2, the improvement was less evident. This confirms the fundamental role of ACE2 in the defense of our cells from the pro-inflammatory action of PM2.5. However, the important point is that, in WT mice, the exposure to PM2.5 induces a significant increase in ACE2 [2].

In conclusion, ACE2 is important because (a) it activates the Nrf2 (anti-inflammatory) pathway and instead turns off NFKB (inflammatory) to avoid a dysregulated inflammation response; (b) COVID-19, by binding to ACE2, alters this mechanism; and (c) ACE2 is over-expressed following exposure to PM2.5, and thus it may be plausible that it could increase the probability of COVID-19 infection, with ACE2 being the entry key for the virus.

Recently, Asselta et al. [69] have analyzed the possible genetic explanation for the male predisposition to COVID-19 infections; the analysis focuses on two genes, TMPRSS2 and ACE2. The first seems to be an excellent candidate because of the presence of a certain allelic variant in the Italian male population. ACE2, on the other hand, was not related to the sex-predisposition, but a single nucleotide polymorphism (SNP), rs2285666, was detected in the Italian and East Asian population. This SNP had already been analyzed as a potential risk factor for hypertension, type 2 diabetes, and coronary artery problems, and thus could be a genetic variant indicating a comorbidity for the virus. According to the study, ACE2 would play an important role, but it remains to be assessed whether the increase in the level of lung gene expression is related to an increase in the susceptibility and severity of COVID-19. This increase in the level of expression of this variant could be owing to exposure by PM [69].

## 9. Conclusions

These analyses on the PM and COVID-19 correlation are the foundations to start wider research. The correlation between these factors is positive, but it is important to understand the mechanism that explains it. All mentioned studies indicate that both long-term exposure and short-term exposure to high levels of pollutants are correlated to an increase in COVID-19 contagion worldwide.

Thus, it may be interesting to perform a systematic study, placing PM collection units at strategic points all over the world, for example, hospital departments with a low ventilation rate, and to analyze the microorganisms present in the search for COVID-19 viral RNA by metagenomics. Preliminary data obtained by Setti and collaborators [70] would support this hypothesis, as Sars-Cov-2 RNA can be present on 34 outdoor samples collected in industrial site of Bergamo Province, in a three-week period, from 21 February to 13 March 13. On the other hand, the populations (also considering gender, age, and genetic factors) that live in areas at high levels of pollutants are in a chronic inflammatory state, which makes them more susceptible to respiratory and cardiovascular diseases.

Lastly, COVID-19 infection should be investigated in relation to ACE2 expression after PM exposure in order to verify the different susceptibility to infection by PM exposed and non-exposed cells.
